# Novel tropolones induce the unfolded protein response pathway and apoptosis in multiple myeloma cells

**DOI:** 10.18632/oncotarget.18543

**Published:** 2017-06-16

**Authors:** Staci L. Haney, Cheryl Allen, Michelle L. Varney, Kaitlyn M. Dykstra, Eric R. Falcone, Sean H. Colligan, Qiang Hu, Alyssa M. Aldridge, Dennis L. Wright, Andrew J. Wiemer, Sarah A. Holstein

**Affiliations:** ^1^ Department of Internal Medicine, University of Nebraska Medical Center, Omaha, NE, USA; ^2^ Department of Medicine, Roswell Park Cancer Institute, Buffalo, NY, USA; ^3^ Department of Pharmaceutical Sciences, University of Connecticut, Storrs, CT, USA; ^4^ Biostatistics and Bioinformatics, Roswell Park Cancer Institute, Buffalo, NY, USA; ^5^ Brown University, Providence, RI, USA

**Keywords:** myeloma, tropolone, histone deacetylase, unfolded protein response, apoptosis

## Abstract

Tropolones are small organic compounds with metal-directing moieties. Tropolones inhibit the proliferation of cancer cell lines, possibly through their effects on metalloenzymes such as select histone deacetylases (HDACs). Pan-HDAC inhibitors are therapeutically beneficial in the treatment of multiple myeloma, however there is interest in the use of more selective HDAC inhibitor therapy to minimize adverse side effects. We hypothesized that tropolones might have anti-myeloma activities. To this end, a series of novel α-substituted tropolones were evaluated for effects on multiple myeloma cells. While all tested tropolones showed some level of cytotoxicity, MO-OH-Nap had consistently low IC_50_ values between 1–11 μM in all three cell lines tested and was used for subsequent experiments. MO-OH-Nap was found to induce apoptosis in a concentration-dependent manner. Time course experiments demonstrated that MO-OH-Nap promotes caspase cleavage in a time frame that was distinct from the pan-HDAC inhibitor suberoylanilide hydroxamic acid (SAHA). Furthermore, MO-OH-Nap- and SAHA-treated cells possess unique gene expression patterns, suggesting they promote apoptosis via different mechanisms. In particular, MO-OH-Nap increases the expression of markers associated with endoplasmic reticulum stress and the unfolded protein response. Synergistic cytotoxic effects were observed when cells were treated with the combination of MO-OH-Nap and the proteasome inhibitor bortezomib. However, treatment with MO-OH-Nap did not abrogate the bortezomib-induced increase in aggresomes, consistent with an HDAC6-independent mechanism for the observed synergy. Collectively, these finding support further investigation into the usefulness of α-substituted tropolones as anti-myeloma agents.

## INTRODUCTION

**Figure 1:**
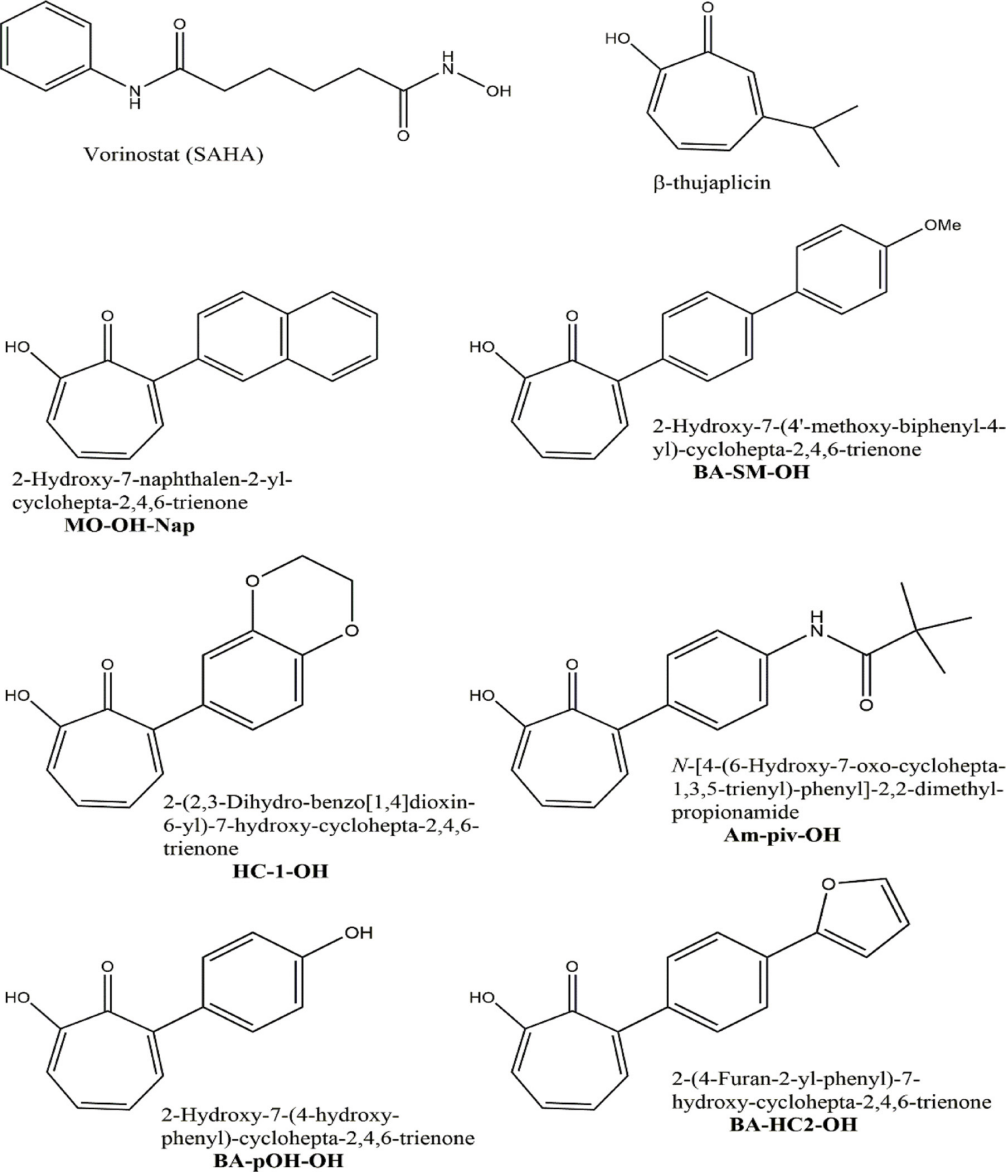
Chemical structures of the pan-HDAC inhibitor SAHA, the parent tropolone β-thujaplicin, and the novel α-substituted tropolone derivatives used in this study

Tropolones are naturally occurring seven-membered non-benzenoid aromatic compounds that are capable of chelating metal ions. β-thujaplicin (Figure 1) is a representative tropolone that is found in several plants of the Cupressaceae family. Functionally, β-thujaplicin possesses anti-fungal, anti-bacterial, and antioxidant properties [1–3]. In addition, many tropolone derivatives have been shown to possess anti-proliferative properties in various cancer cell lines, including lung, prostate and T-cell malignancies [4–7]. Unlike many natural products, tropolones have a low molecular weight and a relatively simple backbone that allows for extensive structural modification. These key characteristics, along with their limited toxicity in normal cells, make them ideal scaffolds for drug development [4, 7]. Despite their simple structure, tropolones are functionally unique and possess a hydroxyketone in their aromatic ring that permits binding to metal ions, allowing them to function as both metal chelators and possible metalloenzyme inhibitors [8–12].

It was previously demonstrated that some tropolones can inhibit select histone deacetylases (HDACs), including HDAC2 and HDAC8 [13]. Targeting histone acetylation in cancer cells can lead to reprogramming of the epigenetic state and alterations in gene expression that make the cells more vulnerable to killing. Identification of novel, more selective HDAC inhibitors could prove clinically useful. Currently, pan-HDAC inhibitors are used for the treatment of select hematological malignancies. For example, vorinostat (suberoylanilide hydroxamic acid, SAHA) and romidepsin are used to treat cutaneous T-cell lymphoma [14]. Another pan-HDAC inhibitor, panobinostat, has been approved for use in combination with the proteasome inhibitor bortezomib in relapsed/refractory multiple myeloma [15]. In addition, several clinical trials have demonstrated the activity of panobinostat when used in combination with other standard myeloma therapies, including carfilzomib (a proteasome inhibitor) and thalidomide (an immunomodulatory agent) [16, 17]. The clinically utilized pan-HDAC inhibitors are associated with a variety of side effects, which may in part be due to their lack of specificity with respect to HDACs.

As pan-HDAC inhibitors have proven beneficial in the treatment of myeloma, we hypothesized that tropolones would have cytotoxic affects in myeloma cells but in a manner which may be dissimilar from the pan-HDAC inhibition. To investigate the activity of tropolones in multiple myeloma cells, a library of six novel α-substituted tropolones (Figure 1) were tested for cytotoxic activity in multiple myeloma cells. Amongst the tested tropolones, MO-OH-Nap had the most consistent cytotoxic effects and was used to further dissect the anti-proliferative effect of tropolones in myeloma cells. These studies reveal that the novel tropolone MO-OH-Nap induces the unfolded protein response pathway and apoptosis in myeloma cells.

## RESULTS

### The novel α-substituted tropolones induce cytotoxic effects in myeloma cell lines

**Figure 2:**
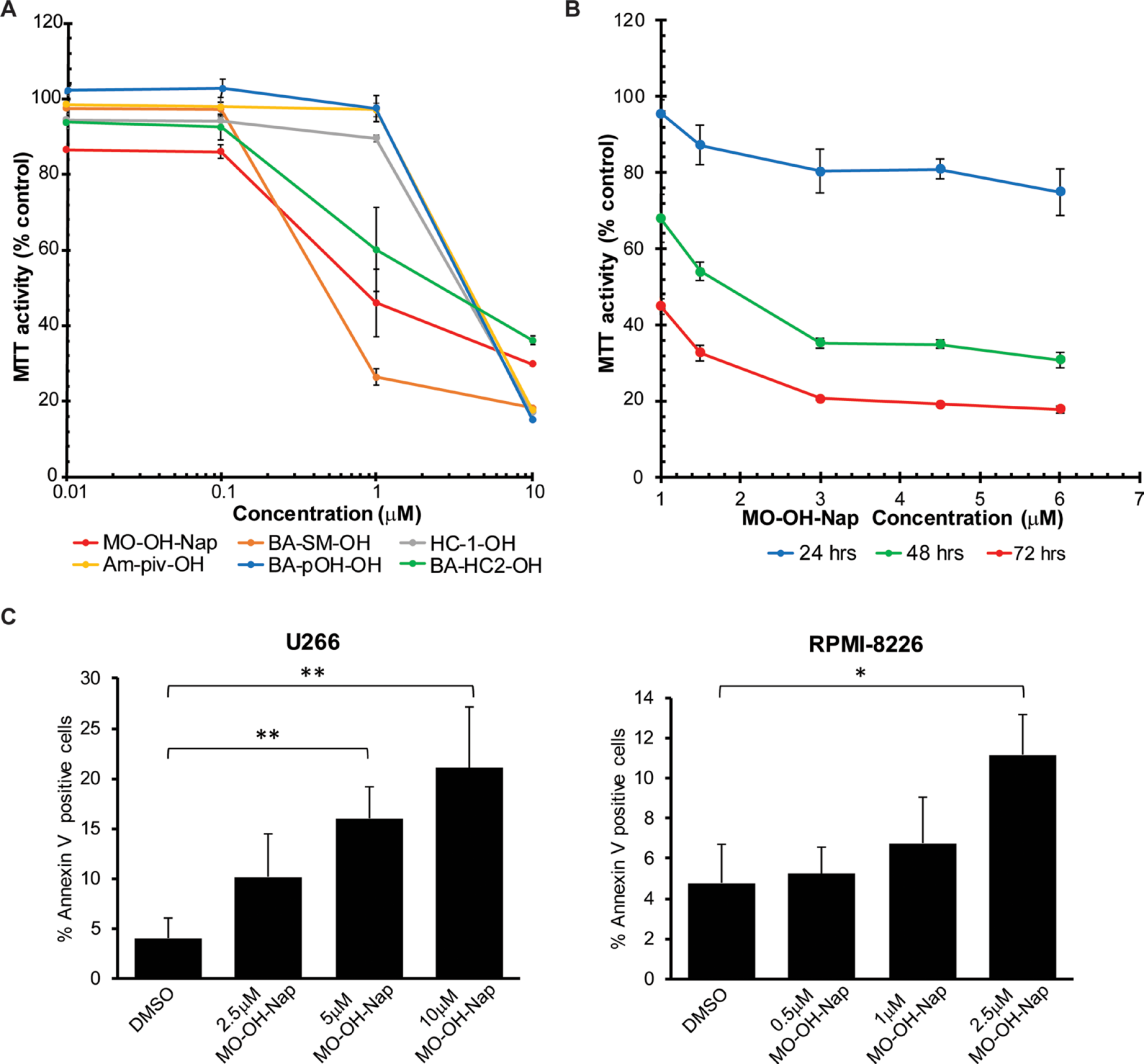
The novel tropolones induce cytotoxic effects in myeloma cells (**A**) MTT assays were performed following a 48-hour incubation period with the tropolones in RPMI-8226 cells. (**B**) Time-dependent effects of MO-OH-Nap in RPMI-8226 cells. Data are expressed as percentage of control (mean + standard deviation, *n* = 4). (**C**) U266 and (**D**) RPMI-8226 cells were treated with DMSO (solvent control) or MO-OH-Nap for 48 hours. Cells were stained with fluorescently conjugated Annexin V and 7-AAD and analyzed by flow cytometry. Data are expressed as the average percentage of Annexin V-positive, 7-AAD-negative cells (*n* = 3, error bars denote standard deviation, *denotes *p* < 0.05. **denotes *p* < 0.01).

The six novel α-substituted tropolones (Figure 1) were tested for cytotoxic activity using an MTT assay in three myeloma cell lines (RPMI-8226, U266, and MM.1S). As shown in Table 1, the patterns of activity in the various cell lines were similar amongst the tropolones such that RPMI-8226 cells were the most sensitive, MM.1S intermediate, and U266 cells least sensitive. In general, the tropolones with more polar substituents were less potent (Table 1, Figure 2A). Amongst the tropolones, MO-OH-Nap had the most consistent cytotoxic effects across the tested cell lines, thus this agent was chosen for all subsequent studies. The cytotoxic effects of MO-OH-Nap increased over time in a concentration-dependent manner (Figure 2B, Supplementary Figure 1). MO-OH-Nap also induced a concentration-dependent decrease in cell proliferation as measured by a BrdU incorporation assay (Supplementary Figure 2). The ability of MO-OH-Nap to inhibit proliferation was not affected by co-incubation of the myeloma cells with HS-5 bone marrow stromal cells (Supplementary Figure 2). Likewise, Annexin V/7-AAD flow cytometric studies demonstrated that MO-OH-Nap treatment promotes apoptosis in a concentration-dependent manner in RPMI-8226 and U266 cells (Figure 2C, 2D and Supplementary Figure 3).

**Table 1:** Cytotoxic activity of the novel α-substituted tropolones

	MTT assay IC_50_ (μM) (48-hour incubation)		
	RPMI-8226	U266	MM.1S
MO-OH-Nap	1.2	11	10
HC-1-OH	2.4	89	24
BA-SM-OH	0.5	68	3.9
BA-pOH-OH	4.4	> 100	68
Am-piv-OH	2.2	> 100	23
BA-HC2-OH	2.2	18	6.8
SAHA	1.01	2.96	0.93

IC_50_ values for myeloma cells treated with various tropolone derivatives or SAHA are shown.

### MO-OH-Nap induces caspase cleavage

**Figure 3:**
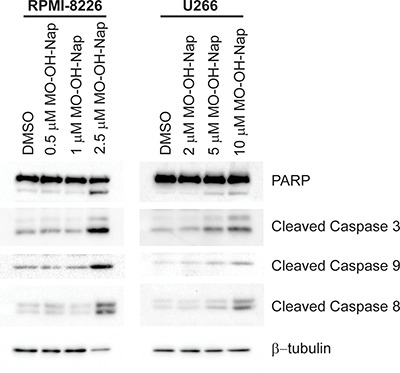
The novel tropolone MO-OH-Nap induces apoptosis in a concentration-dependent manner RPMI-8226 and U266 were incubated in the presence or absence of MO-OH-Nap or solvent control (DMSO). Whole cell lysate was obtained and immunoblot analysis of PARP, cleaved caspases-3, -8 and -9 was performed along with β-tubulin as a loading control. The gels are representative of three independent experiments.

**Figure 4:**
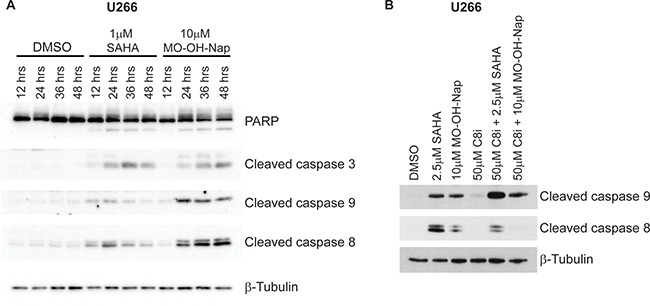
MO-OH-Nap induces caspase cleavage in a time-dependent manner which is distinct from the pan-HDAC inhibitor SAHA (**A**) U266 cells were incubated for 12-48 hours in the presence or absence of SAHA (1 μM) or MO-OH-Nap (10 μM). Whole cell lysate was obtained and immunoblot analysis of PARP, cleaved caspases-3, -8 and -9 was performed along with β-tubulin as a loading control. The gels are representative of three independent experiments. (**B**) U266 cells were incubated for 24 hours in the presence or absence of a caspase-8 inhibitor (Z-IETD-FMK, *C8i,* 50 μM) and/or SAHA (2.5 μM) or MO-OH-Nap (10 μM). Whole cell lysate was obtained and immunoblot analysis of cleaved caspases-8 and -9 was performed along with β-tubulin as a loading control. The gels are representative of three independent experiments.

To more directly assess the ability of MO-OH-Nap to induce apoptosis in myeloma cells, immunoblot analysis was performed to detect cleaved caspases 3, 9, and 8. As shown in Figure 3, treatment with MO-OH-Nap for 48 hours results in the cleavage of these caspases in a concentration-dependent manner in both RPMI-8226 and U266 cells. Next, time-course studies were performed to determine the timing of the MO-OH-Nap-induced caspase cleavage and to determine whether this pattern differed from the pan-HDAC inhibitor SAHA. As shown in Figure 4A, SAHA induced maximal caspase 8 and 9 cleavage by 24 hours, and caspase 3 cleavage by 36 hours in U266 cells. In contrast, maximal cleavage of these caspases occurred by 36–48 hours following tropolone treatment. Co-incubation with Z-IETD-FMK, a specific caspase 8 inhibitor, prevented both SAHA- and MO-OH-Nap-induced caspase 8 cleavage but did cause an increase in caspase 9 cleavage (Figure 4B).

### MO-OH-Nap and SAHA induce distinct patterns of gene expression

**Figure 5:**
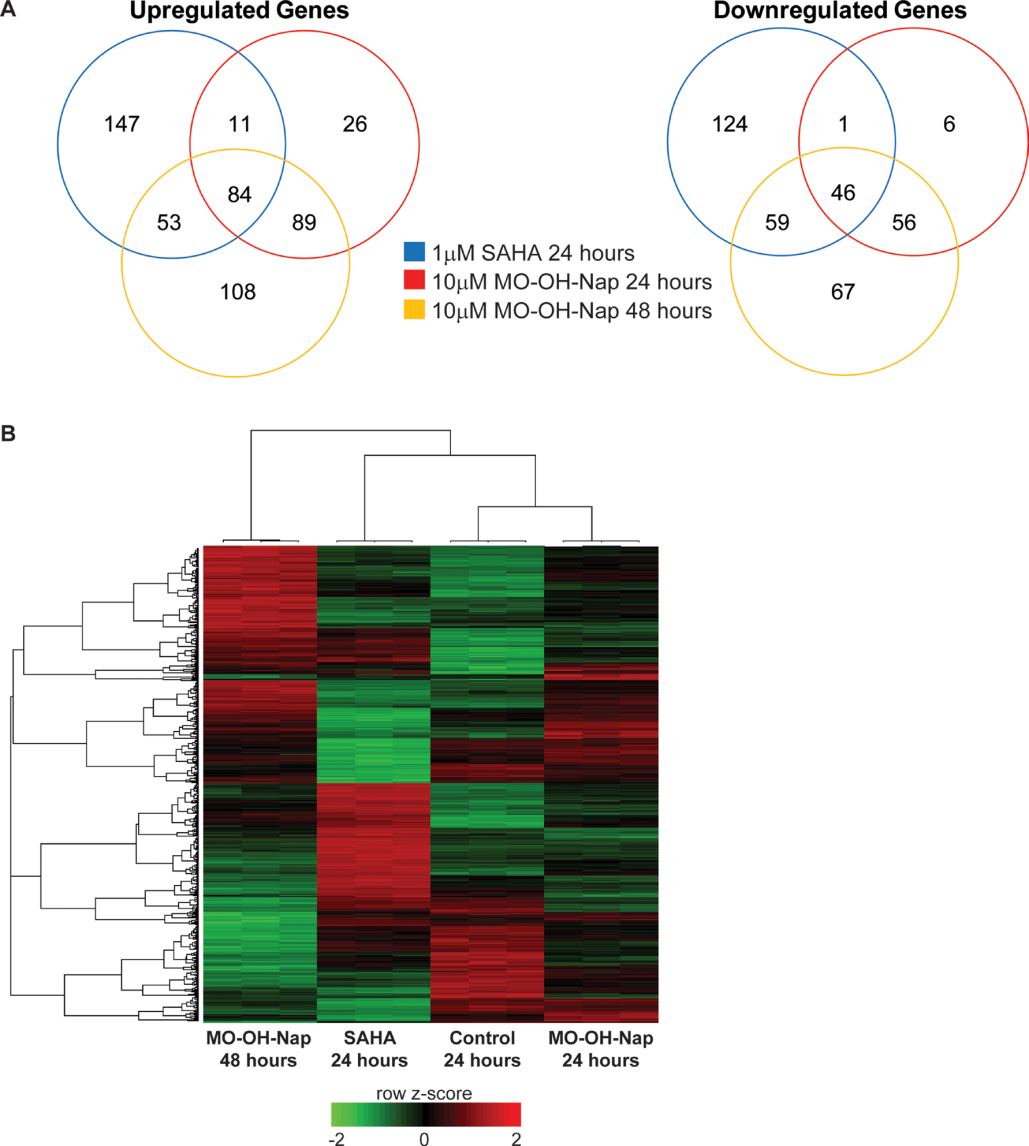
MO-OH-Nap and SAHA induce distinct patterns of gene expression (**A**) Venn diagrams showing overlaps in gene expression for upregulated and downregulated genes in SAHA-24 hour (blue), MO-OH-Nap-24 hour (red) and MO-OH-Nap-48 hour (yellow) treated cells relative to the untreated control. (**B**) Heatmap of differentially expressed in the four treatment groups. Unsupervised hierarchical clustering is shown for individual genes (left) and samples (top). The color key shows row z-scores in which red denotes highly expressed genes and green denotes lowly expressed genes.

The differences in timing of caspase cleavage induced by MO-OH-Nap and SAHA suggested disparate mechanisms of action. To further explore this, microarray studies were performed using RNA isolated from U266 cells treated with either SAHA or MO-OH-Nap. Expression profiles generated from U266 cells treated either with SAHA for 24 hours, MO-OH-Nap for 24 hours or MO-OH-NAP for 48 hours were compared to untreated control cells. All three treatment groups had diverse gene expression patterns, with SAHA-24 hour and MO-OH-Nap-48 hour producing the highest number of distinct gene expression changes (Figure 5A). In total, 84 upregulated and 46 downregulated genes were shared between all three treatment groups, suggesting some downstream events are likely shared (Figure 5A). Hierarchical clustering resulted in separation of samples into treatment groups and a heatmap of differentially expressed genes further illustrated the distinctive gene expression patterns of the four groups (Figure 5B).

Next, we used Ingenuity Pathway Analysis to investigate the deregulated molecular pathways that occur as a result of SAHA and MO-OH-Nap treatment. The top pathway hits for the SAHA group were largely related to cell cycle control, while upstream analysis suggested activation of both p53 and p21, and inhibition of Myc (Table 2 and Supplementary Table 2). One pathway that was noteworthy was the unfolded protein response (UPR) pathway. IPA analysis revealed that the UPR and ER stress pathways were both top hits in the MO-OH-Nap 48 hour treated cells, however neither were predicted to be activated in the SAHA-treated group (Table 2). Furthermore, in the 48-hour MO-OH-Nap-treated group upstream analysis predicted activation of XBP-1 and ATF6, both of which are central regulators of the UPR (Supplementary Table 2).

**Table 2:** Ingenuity pathway analysis: Top canonical pathways

*MOOH-24 hr vs Control*		
Pathway	*p*-value	Overlap
Glycolysis I	4.98E-10	32.0% (8/25)
Gluconeogenesis I	1.80E-08	28.0% (7/25)
Unfolded protein response	3.50E-07	14.8% (8/54)
Sucrose Degradation V (Mammalian)	1.55E-04	33.3% (3/9)
Cysteine Biosynthesis/Homocysteine Degradation	1.57E-04	100.0% (2/2)
*MOOH-48 hr vs Control*		
**Pathway**	***p*-value**	**Overlap**
Unfolded protein response	6.53E-12	25.9% (14/54)
Endoplasmic Reticulum Stress Pathway	1.99E-07	33.3% (7/21)
Antigen Presentation Pathway	1.36E-04	16.2% (6/37)
Estrogen-mediated S-phase Entry	1.45E-04	20.8% (5/24)
Glucocorticoid Receptor Signaling	2.03E-04	5.9% (17/287)
*Saha-24 hr vs Control*		
**Pathway**	***p*-value**	**Overlap**
Cell Cycle Control of Chromosomal Replication	6.41E-07	21.1% (8/38)
Estrogen-mediated S-phase Entry	5.78E-06	5.0% (6/24)
Germ Cell-Sertoli Cell Junction Signaling	4.28E-05	7.5% (13/173)
Actin Nucleation by ARP-WASP Complex	1.14E-04	12.5% (7/56)
Cyclins and Cell Cycle Regulation	1.54E-04	10.3% (8/78)

Differentially expressed genes (DEG) were analyzed using Ingenuity Pathway Analysis. The top hit for “Canonical Pathways” are shown, as well as the associated *p*-value and number of DEG that overlap with each pathway. A *p*-value < 0.05 was considered significant.

### MO-OH-Nap induces markers of ER stress and the UPR

**Figure 6:**
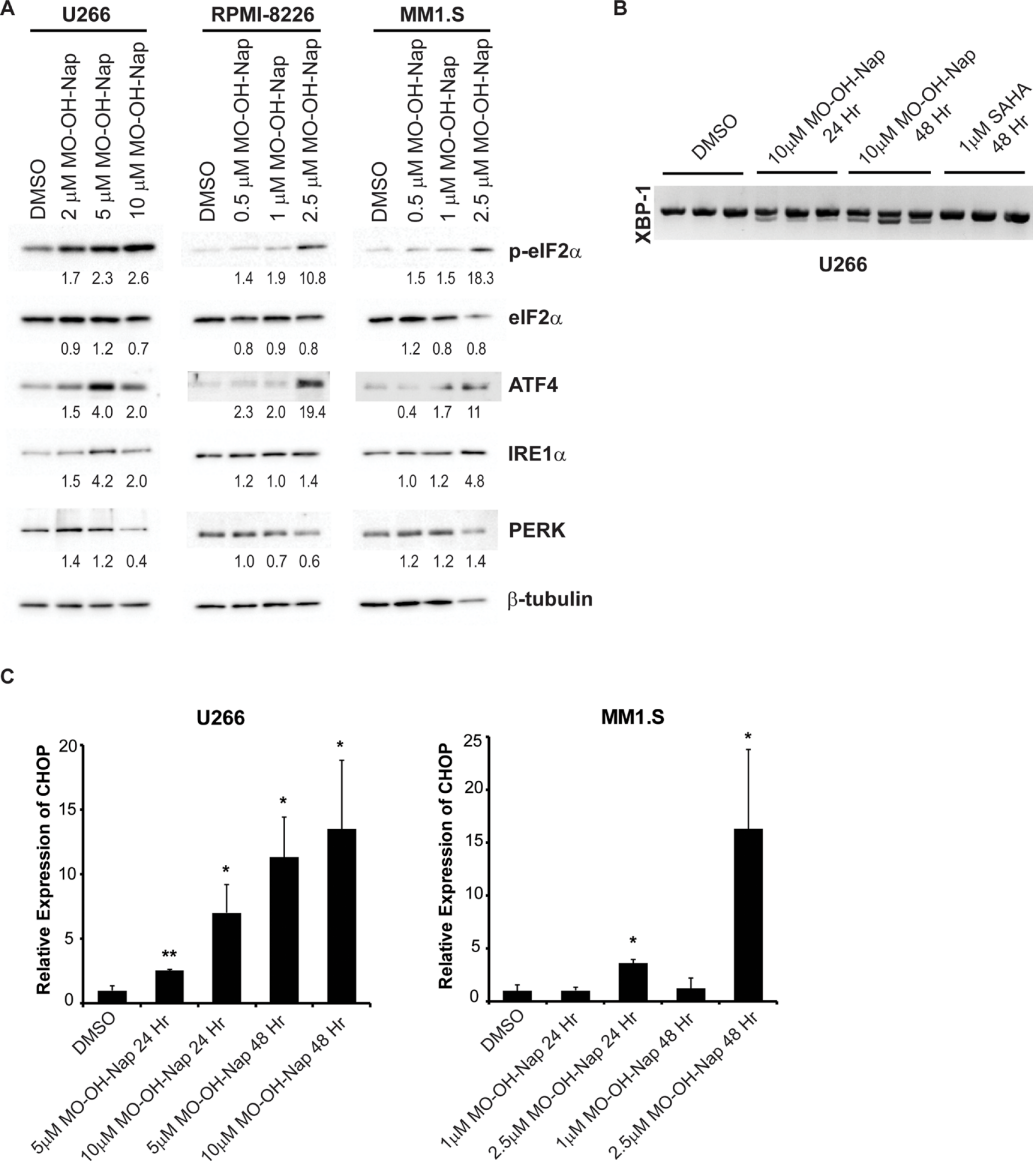
MO-OH-Nap induces markers of the unfolded protein response pathway (**A**) U266, RPMI-8226, and MM1.S cells were incubated in the presence or absence of DMSO (solvent control) or MO-OH-Nap for 48 hours. Immunoblot analysis of phosphorylated eIF2α (p-eIF2α), eIF2α, ATF4, IRE1α, and PERK was performed along with β-tubulin as a loading control. Densitometric analysis of UPR proteins (normalized to β-tubulin) for the treated cells normalized to DMSO (solvent control) cells is shown. (**B**) U266 cells were incubated for 24 or 48 hours with solvent control, SAHA, or MO-OH-Nap in triplicate. RNA was isolated, cDNA prepared, and PCR was performed using XBP-1-specific primers. The upper band represents unspliced XBP-1 and the lower band represents spliced XBP-1. (**C**) qRT-PCR analysis of CHOP expression in U266 and MM1.S cells incubated in the presence or absence of DMSO (solvent control) or MO-OH-Nap for 24 or 48 hours. Data represents fold change normalized to DMSO control. (*n* = 3 independent experiments, error bars denote standard error, *denotes *p* < 0.05. **denotes *p* < 0.01).

To verify the microarray studies that had revealed that multiple genes involved in ER stress and the UPR were upregulated following treatment with MO-OH-Nap, immunoblot studies were performed. As shown in Figure 6A, treatment with MO-OH-Nap results in an increase in activating transcription factor 4 (ATF4), inositol-requiring enzyme 1α (IRE1α), PRLR-like ER kinase (PERK), and phosphorylated eukaryotic translation initiation factor 2-α (eIF2α). Furthermore, MO-OH-Nap-induced splicing of X-box binding protein-1 (XBP-1), whereas SAHA did not (Figure 6B and Supplementary Figure 4). In addition, qRT-PCR analysis showed upregulation of CHOP mRNA in MO-OH-Nap treated cells at 24 and 48 hours (Figure 6C). Furthermore, protein levels of acetylated histones, H3K9, H3K23, and H4K8, were increased by treatment with MO-OH-Nap in a concentration-dependent manner, suggesting disruption of HDAC activity (Supplementary Figure 5). The induction of ATF4 by MO-OH-Nap preceded the induction of caspase cleavage (Supplementary Figure 6, Figure 4A) and, consistent with the microarray studies, SAHA did not induce ATF4 expression (Supplementary Figure 6). Another tropolone, BA-HC2-OH, exerted similar effects on markers of apoptosis and the UPR (Supplementary Figure 7).

### MO-OH-Nap and bortezomib induce synergistic cytotoxic effects

**Figure 7:**
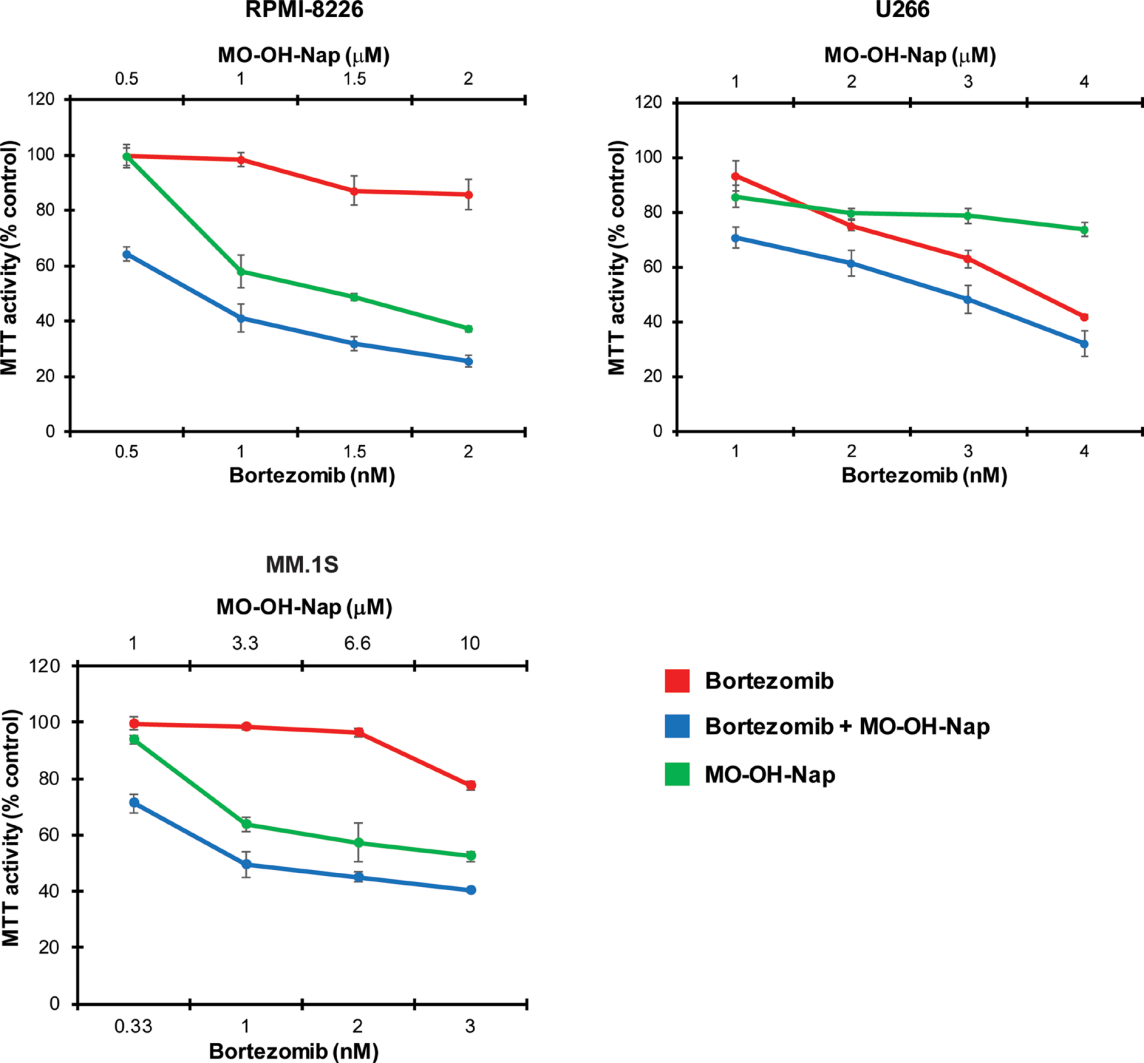
The combination of bortezomib and MO-OH-Nap induces synergistic cytotoxic effects in myeloma cells MTT assays were performed following the incubation of cells with bortezomib and/or MO-OH-Nap for 48 hours. Data are expressed as percentage of control (mean ± standard deviation, *n* = 4).

Prior studies investigating the combination of pan-HDAC inhibitors and proteasome inhibitors have revealed synergistic cytotoxic effects [18–21]. MTT cytotoxicity studies were therefore performed to determine whether the novel tropolone MO-OH-Nap is synergistic with the proteasome inhibitor bortezomib. As shown in Figure 7, synergistic interactions, as defined by a combination index of < 1 (as per Chou and Talalay [22]), were observed between the two agents (Supplementary Table 3). Primarily additive or synergistic interactions were also observed when MO-OH-Nap was used in combination with the mevalonate pathway inhibitor lovastatin, which we have previously demonstrated induces the UPR via disruption of protein trafficking [23, 24] (Supplementary Figure 8, Supplementary Table 3).

**Figure 8:**
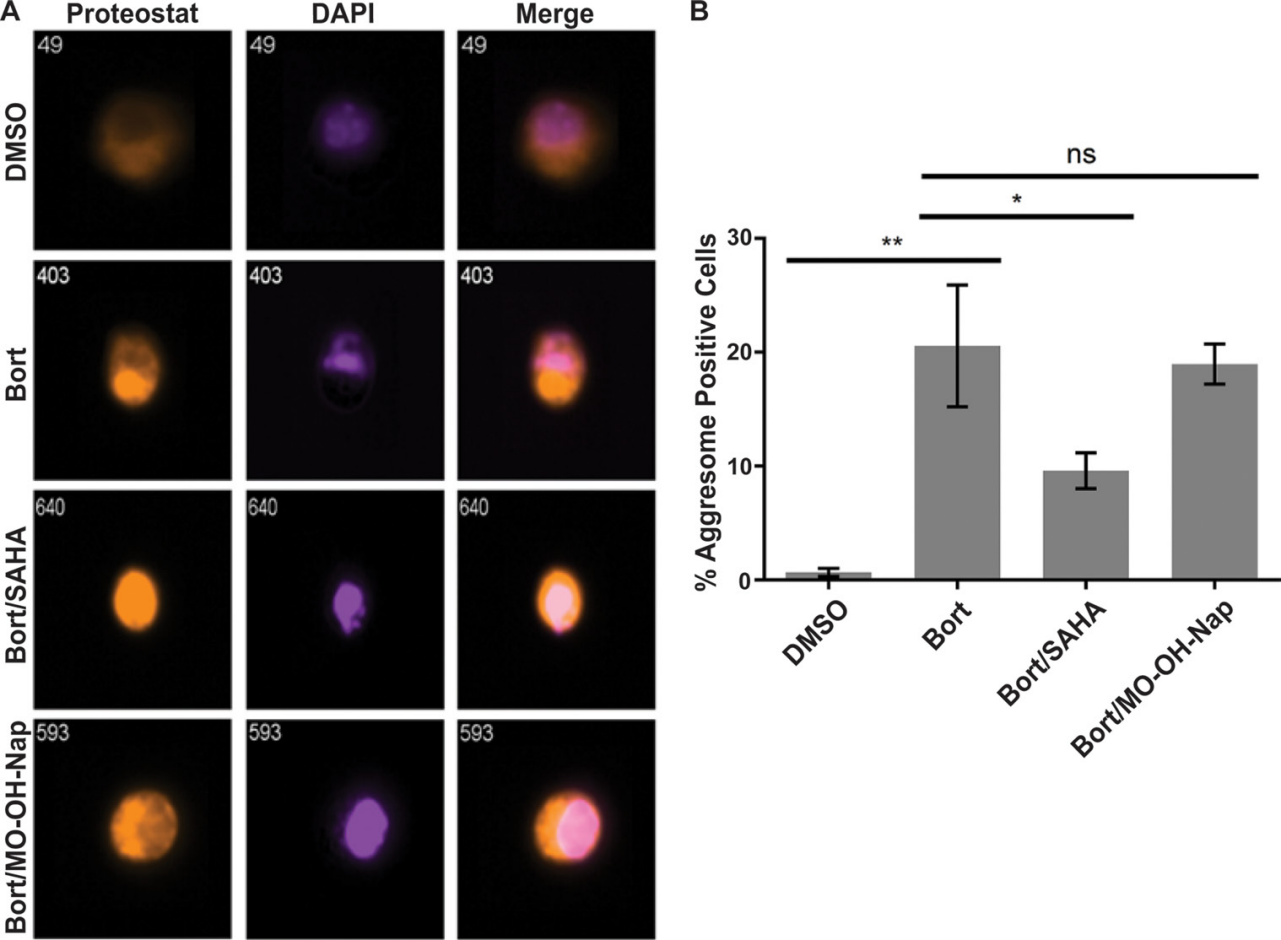
MO-OH-Nap does not impact bortezomib-induced aggresome formation U266 cells were treated for 24 hours with 7.5 nM bortezomib (*Bort*) with or without 1 μM SAHA or 10 μM MO-OH-Nap. Aggresome staining was performed as described in the Materials and Methods. Cells were gated as aggresome-positive for fluorescence intensities that were low Proteostat intensity in the nucleus and high Proteostat intensity outside the nucleus, indicating a perinuclear aggresome versus aggregates dispersed throughout the cell. Representative cells are shown in (**A**) and the analyzed data in (**B**) (*N* = 3 independent experiments), * indicates *p* < 0.05, **indicates *p* < 0.01.

The synergy between HDAC inhibitors and proteasome inhibitors is at least in part explained by HDAC6 inhibition which abrogates aggresome formation, thus inhibiting the degradation of misfolded proteins leading to their accumulation within cytoplasm and further activation of pro-apoptotic events [25]. The induced increase in aggresomes following bortezomib treatment is partially prevented by co-treatment with SAHA. However, treatment with MO-OH-Nap has no effect on the bortezomib-induced increase in aggresomes, consistent with a mechanism of action that is HDAC6-independent (Figure 8 and Supplementary Figure 9). In addition, MO-OH-Nap did not alter the levels of acetylated alpha-tubulin in myeloma cell lines, further suggesting that HDAC6 activity is not altered (Supplementary Figure 10).

## DISCUSSION

Myeloma cells are highly secretory and there has been considerable effort focused on drug development strategies which take advantage of the reliance on protein homeostasis mechanisms including synthesis, degradation, and trafficking. More recently, pan-HDAC inhibitor therapy has been added to the armamentarium of proteasome inhibitors and immunomodulatory drugs which are most commonly used to treat myeloma [26, 27]. Currently under development are HDAC-6-specific inhibitors, as synergy has been observed with proteasome inhibitors [28, 29]. Selective tropolone analogues have previously been shown to have effects on histone acetylation and to have selective cytotoxic effects for malignant cells compared to healthy human peripheral blood mononuclear cells [6, 13]. Here we have demonstrated that MO-OH-Nap, a novel α-substituted tropolone, induces apoptosis and the UPR in myeloma cells.

We found that MO-OH-Nap treatment inhibits proliferation and induces caspase cleavage in myeloma cells, consistent with previous studies in leukemia cell lines where α-tropolone derivatives promoted apoptosis through caspase activation in a concentration-dependent manner [6]. Both the classic intrinsic (caspases 9 and 3) and extrinsic (caspases 8 and 3) apoptotic pathways are involved. The time-line of caspase cleavage observed in MO-OH-Nap treated cells differs from that observed with SAHA, which induced maximal caspase cleavage around 24 hours. While both agents are capable of inducing apoptosis in myeloma cells, the difference in timing of caspase cleavage suggests that the two compounds have different mechanisms of action. While earlier studies showed that some tropolone derivatives function as selective inhibitors of HDACs [13], more recent work has suggested that the effect on histone acetylation was dependent on caspase activation [4]. IPA analysis of our microarray data indicates that HDAC activity is disrupted in both SAHA and MO-OH-Nap treated cells and, for MO-OH-Nap, at a time point which precedes maximal induction of caspase cleavage.

The dissimilar phenotypes observed upon treatment with SAHA and MO-OH-Nap were supported by the microarray data where we observed unique gene expression profiles. Analysis of microarray data using IPA software reveal that SAHA-treated myeloma cells respond in a way that is similar to other studies involving SAHA, including p53 and p21 activation, MYC inhibition, and dysregulation of pathways related to cell cycle [6, 7, 30–33]. While IPA revealed there were some similarities between the MO-OH-Nap- and SAHA-treated cells, such as p53 inhibition, several unique characteristics were observed. The two pathways with the lowest p-value in the MO-OH-Nap treated cells at 48 hours were the UPR and ER stress pathways. Consistent with these results, we observed activation of XBP-1 by alternative mRNA splicing and upregulation of markers associated with ER stress, such as ATF4, IRE1α, PERK, and phosphorylated eIF2a, in MO-OH-Nap-treated cells. Furthermore, when 24-hour MO-OH-Nap treatment was compared to 48-hour, the UPR and ER stress pathways were again the highest hits, suggesting that over the 24-hour period MO-OH-Nap is promoting ER stress and activation of the UPR, which in turn leads to caspase cleavage and apoptosis in myeloma cells. Additional investigation will be needed to fully understand the mechanism by which MO-OH-Nap induces ER stress and the UPR and to determine whether the observed differences in sensitivity of the tested myeloma cell lines to MO-OH-Nap corresponds to known differences in factors such as p53 status or whether other factors are involved. Of note, select class I HDACs, including HDACs 1, 2, and 3 have been shown to localize to the ER and to interact with the key chaperone protein GRP78 which is involved with regulation of the UPR [34]. In addition, the silencing of class I HDACs resulted in activation of the PERK and ATF6 arms of the UPR, but not the IRE1α arm [34]. However, our studies demonstrate the activation of all three arms of the UPR, thus suggesting that there may be HDAC-independent effects of the tropolone. Whether the iron-binding ability of tropolones [6, 10–12] is involved in the mechanisms underlying the observed induction of the UPR remains to be determined.

Another interesting finding that came out of this study was the synergistic effect observed between MO-OH-Nap and the proteasome inhibitor bortezomib. Because myeloma cells are continuously secreting large quantities of monoclonal protein, they are particularly sensitive to interruption of routine proteolysis, and proteasome inhibitor therapy is widely used in both the newly diagnosed and relapsed/refractory setting for myeloma. However, because many myeloma patients suffer from relapse due to drug resistance, it is critical to identify new drugs that can overcome resistance mechanisms. Synergy between proteasome and HDAC inhibitors in myeloma has been documented [18–20], and at least in part is due to HDAC6 inhibition and disruption of aggresome formation [25]. However, in our studies, aggresome formation was not altered by the addition of the tropolone, suggesting an HDAC6-independent mechanism for the observed synergy. We predict the synergism is a result of the tropolone's ability to induce ER stress and activation of the UPR leading to enhanced activation of the caspase cascade and apoptosis.

In summary, we have shown that the novel α-substituted tropolone MO-OH-Nap is cytotoxic against myeloma cells and functions in a manner that is distinct from the pan-HDAC inhibitor SAHA. These finding are relevant as they demonstrate a novel mechanism of tropolone activity in myeloma cells. We provide evidence that tropolone treatment induces ER stress and activation of the UPR. Furthermore, MO-OH-Nap was synergistic with the proteasome inhibitor bortezomib, suggesting that a combination therapy may also be beneficial *in vivo*. Future studies are needed to better understand the molecular mechanisms of these novel tropolones to determine their potential as anti-myeloma agents.

## MATERIALS AND METHODS

### Reagents

The tropolones were synthesized as per the previously published methodology [6]. Stock solutions of the tropolones were prepared in DMSO (10 mM) and stored at −20°C. Suberoylanilide hydroxamic acid (SAHA), bortezomib, and lovastatin were purchased from Sigma-Aldrich (St Louis, MO, USA). The caspase-8 inhibitor Z-IETD-FMK was purchased from R&D Systems (Minneapolis, MN, USA).

### Cell culture

Human myeloma (RPMI-8226, MM.1S, U266) and bone marrow stromal (HS-5) cells were purchased from American Type Culture Collection (ATCC) (Manassas, VA, USA). Cells were grown in media (per ATCC specifications) supplemented with heat-inactivated fetal bovine serum (FBS), glutamine and penicillin-streptomycin at 37°C and 5% CO_2_.

### Immunoblotting

Following incubation with drugs, cells were collected, washed with PBS, and lysed in RIPA buffer (0.15M NaCl, 1% sodium deoxycholate, 0.1% SDS, 1% Triton (v/v) X-100, 0.05 M Tris HCl, pH 7.4) containing protease and phosphatase inhibitors. Protein content was determined using the bicinchoninic acid (BCA) method. Equivalent amounts of cell lysate were resolved by SDS-PAGE, transferred to polyvinylidene difluoride membrane, probed with the appropriate primary antibodies, and detected using HRP-linked secondary antibodies and Amersham Pharmacia Biotech ECL Western blotting reagents per manufacturer's protocols. Supplementary Table 1 details the primary and secondary antibodies.

### MTT assay

Cells were seeded (2.5 × 10^4^ cells/100 μL per well) in 96-well flat-bottom plates and incubated with drugs. The MTT assay was performed as previously described [35]. The absorbance for control cells treated with solvent only was defined as an MTT activity of 100%.

### BrdU assays

Cells were plated in 96-well tissue culture plates at 2.5 × 10^4^ cells/100 μL/well (RPMI-8226) or 5 × 10^4^ cells/100 μL/well (U266). Cells were incubated for 48 hours in the presence of solvent control (DMSO) or varying concentrations of MO-OH-Nap. The addition of BrdU occurred 4 (RPMI-8226) or 6 (U266) hours prior to fixation. Plates were spun at 1000 rpm for 10 minutes. Cells were processed per manufacturer's instructions using the Millipore BrdU Cell Proliferation Assay kit. For the co-culture experiments, HS-5 cells were plated in 96-well tissue culture plates at 1 × 10^4^ cells/100 μL/well. After 24 hours, myeloma cells were added to the plate as was solvent control or varying concentrations of MO-OH-Nap. Following 48-hour incubation and then BrDU labeling, the myeloma cells were transferred to a different plate and the plate was processed per the manufacturer's instructions.

### Apoptosis detection studies

U266 or RPMI-8226 cells were treated with solvent control (DMSO) or MO-OH-Nap for 48 hours. Cells were then stained with PE-conjugated Annexin V antibody and the membrane permeability dye 7-AAD according to the manufacturer's recommendations (eBioscience). A total of 20,000 cells were analyzed using a BD LSRII flow cytometer. FlowJo software was used for data analysis. We define early apoptotic cells as those positive for Annexin V and negative for 7-AAD.

### RT-PCR for XBP-1 splicing

Cells were incubated for 24 hours in the presence or absence of solvent control, SAHA, or MO-OH-Nap. Total RNA was isolated, cDNA prepared, and PCR performed using XBP-1-specific primers as described by Yan et al. [36]. PCR products were separated on a 2% agarose gel, stained with ethidium bromide, and visualized on a UV transilluminator. The upper band represents unspliced XBP-1 and the lower band represents spliced XBP-1. Primer sequences can be found in Supplementary Table 4.

### Microarray analysis

U266 cells were treated with solvent control (DMSO), MO-OH-Nap, or SAHA. Following 24 or 48 hour incubations, RNA was extracted using Qiagen RNAEasy kit. Samples were then processed by the Roswell Park Cancer Institute Genomics Shared Resource and analyzed using an Illumina HumanHT-12 v4 Expression BeadChip. Data were analyzed with R language and Bioconductor packages. Microarray data normalization, background correction and quality control were carried out with the Bioconductor package “lumi”. The R package “limma” was used to perform differential gene expression analysis of the normalized microarray data. The significant differentially expressed genes were identified with p value less than 0.05 and fold change with a cut off of 1.5. In order to identify activated and inhibited signaling pathways, all differentially expressed genes were analyzed using Ingenuity pathway analysis (IPA) software (Qiagen). Data files are available for download through NCBI Gene Expression Omnibus (GSE93099).

### Aggresome detection studies

U266 cells were incubated for 24 hours in the absence or presence of drugs. Aggresome staining was performed using Enzo PROTEOSTAT^®^ Aggresome detection kit according to the manufacturer's instructions. Just prior to running on the ImageStream, all samples had DAPI added to visualize the nucleus. In total, 5,000 events were collected for all samples on an ImageStream ISX using 405 nm and 488 nm laser excitation. Cell populations were hierarchically gated for single cells that were in focus and positive for both DAPI and Proteostat. Following data acquisition, masks were created using DAPI for the nucleus (Fill(M07)) and Proteostat for the entire cell (Fill(M04)). The spatial relationship between the Proteostat and nuclear staining was measured by plotting the fluorescence intensity of Proteostat in the nuclear region (Intensity_Fill(M07)_Ch04) versus outside the nuclear region (Intensity_Fill(M04)-Intensity_Fill(M07)_Ch04) Cells were gated as aggresome positive for fluorescence intensities that were low Proteostat intensity in the nucleus and high Proteostat intensity outside the nucleus, indicating a perinuclear aggresome versus aggregates dispersed throughout the cell.

### qRT-PCR

U266 and MM1.S cells were treated with solvent control (DMSO) or MO-OH-Nap for 24 or 48 hours. RNA was isolated using Qiashredder and RNAeasy Plus kits (Qiagen). cDNA was synthesized from 1mg of total RNA using the iScript cDNA synthesis kit (Bio-Rad). iTaq Universal SYBR Green Supermix (Bio-Rad) was mixed with cDNA and gene specific primers at a final volume of 10 μL. qRT-PCR was performed using a CFX96 real time machine (Bio-Rad). All reactions were performed in duplicate and data was normalized to the expression of β-actin. Primer sequences can be found in Supplementary Table 4.

### Statistics

Two-tailed *t*-testing was used to calculate statistical significance. An α of 0.05 was set as the level of significance. Combination indices for the MTT assays were determined via CalcuSyn software (Biosoft) which analyzes drug interactions based on the method of Chou and Talalay [22]. This software was also used to calculate IC_50_ values.

## SUPPLEMENTARY MATERIALS FIGURES AND TABLES


