# Supplementary material for: Novel tropolones induce the unfolded protein response pathway and apoptosis in multiple myeloma cells
Source: Oncotarget. 2017 Jun 16;8(44):76085–98. doi: 10.18632/oncotarget.18543 (PMC5652688; doi:10.18632/oncotarget.18543)
Supplement: Supplementary file 1 [file oncotarget-08-76085-s001.pdf]

Novel tropolones induce the unfolded protein response pathway and apoptosis in multiple myeloma cells

SUPPLEMENTARY MATERIALS

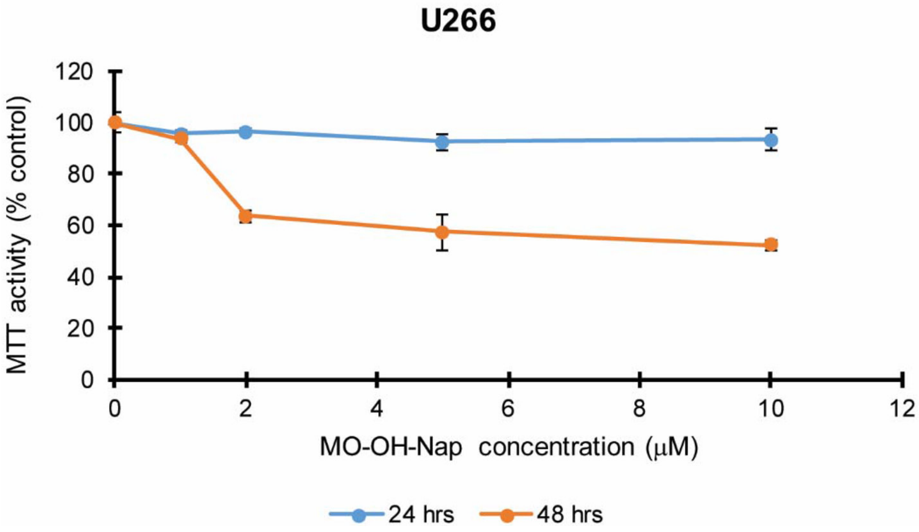

Supplementary Figure 1: Cytotoxic effects of MO-OH-Nap in U266 cells. U266 cells were incubated in the presence or absence of MO-OH-Nap or DMSO (solvent control) for 24 or 48 hours. MTT assays were performed. Data are expressed as percentage of control (mean ± standard deviation, $n = 4$).

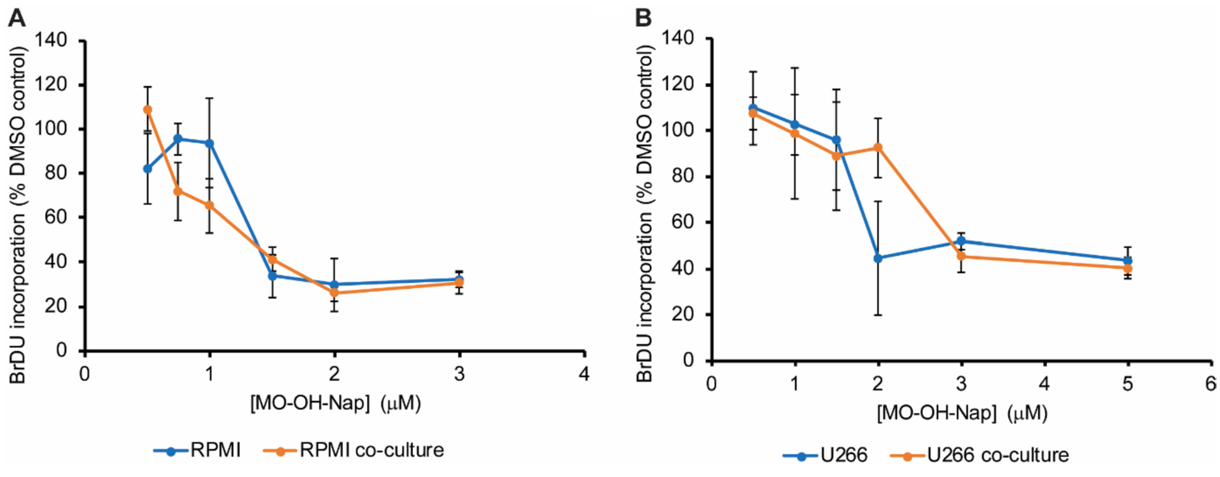

Supplementary Figure 2: MO-OH-Nap treatment slows cellular proliferation in myeloma cell lines. BrdU incorporation was measured following a 48-hour incubation with MO-OH-Nap in RPMI-8226 (**A**) and U266 (**B**) cells cultured alone or in the presence of HS-5 bone marrow stromal cells. Data are expressed as percentage of control (mean ± standard deviation, $n = 4$).

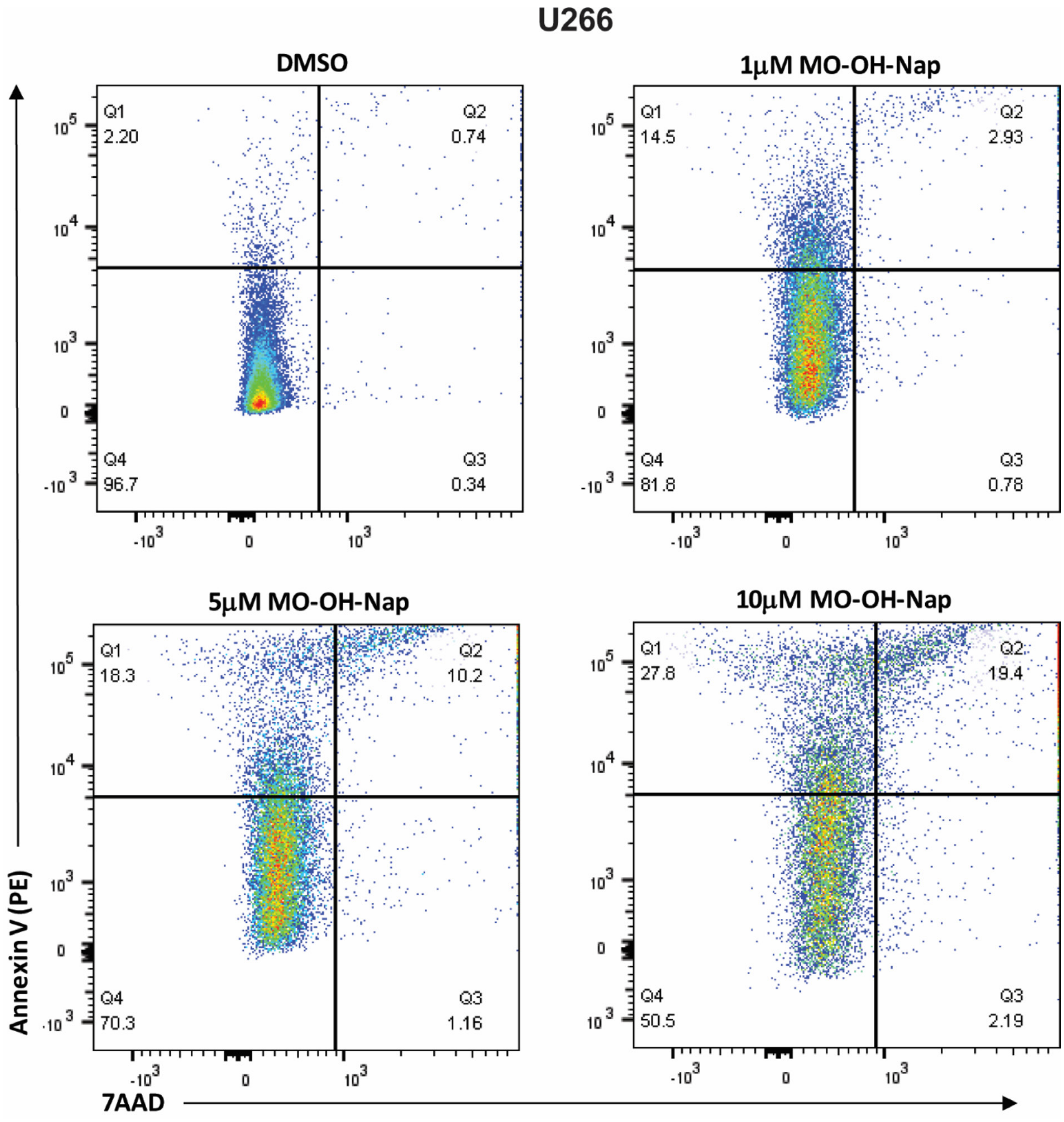

Supplementary Figure 3: Representative example of Annexin V and 7-AAD staining by flow cytometry. U266 cells were treated with DMSO (solvent control) or MO-OH-Nap (1 μM, 5 μM, or 10 μM) for 48 hours and then stained with PE-conjugated Annexin V and 7AAD and analyzed by flow cytometry. The percentage of cells in each quadrant are shown. Quadrant Q1 represents early apoptotic cells, Q2 represents late apoptotic/dead cells, Q3 represents dead cells, and Q4 represents live cells.

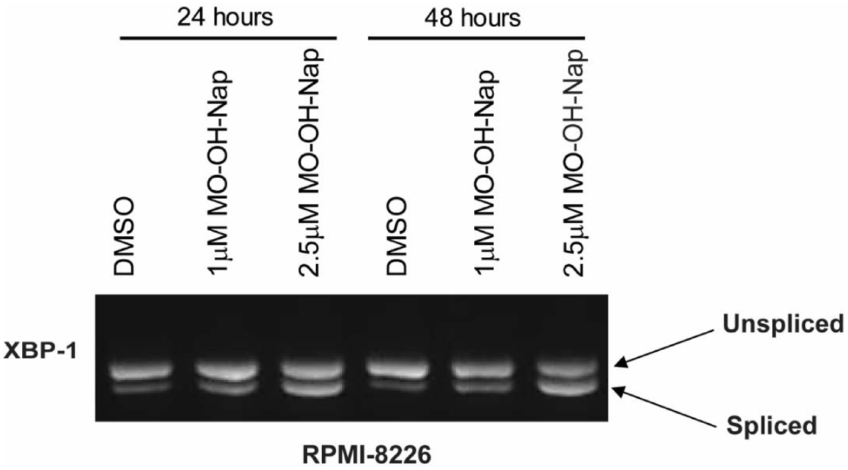

Supplementary Figure 4: MO-OH-Nap induces XBP-1 splicing in RPMI-8266 cells. RPMI-8226 cells were incubated for 24 or 48 hours with DMSO (solvent control) or MO-OH-Nap (1 μM or 2.5 μM). RNA was isolated, cDNA prepared, and PCR was performed using XBP-1-specific primers. The upper band represents unspliced XBP-1 and the lower band represents spliced XBP-1.

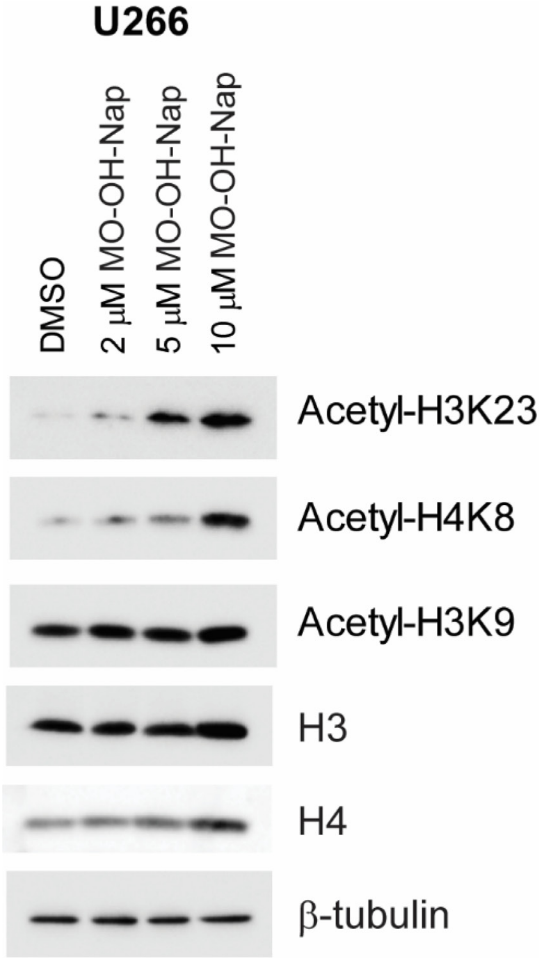

Supplementary Figure 5: Effects of MO-OH-Nap on histone acetylation. U266 cells were incubated for 48 hours in the presence or absence of varying concentrations of MO-OH-Nap. Immunoblot analysis for acetylated H3K23, H4K8, H3K9, total Histone H3, and total Histone H4 was performed as well as β-tubulin as a loading control.

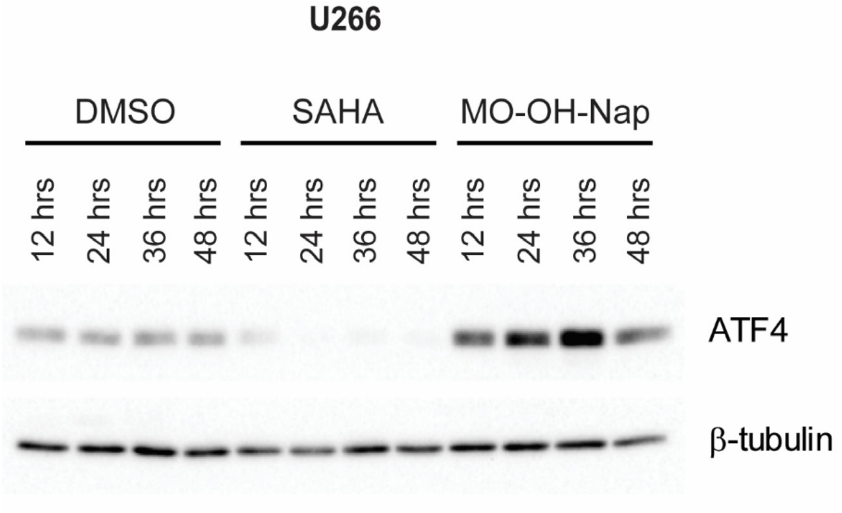

Supplementary Figure 6: MO-OH-Nap induces ATF4 expression. U266 cells were incubated in the presence or absence of SAHA (1 μM) or MO-OH-Nap (10 μM) for 12–48 hours. Immunoblot analysis of ATF4 was performed along with β-tubulin as a loading control.

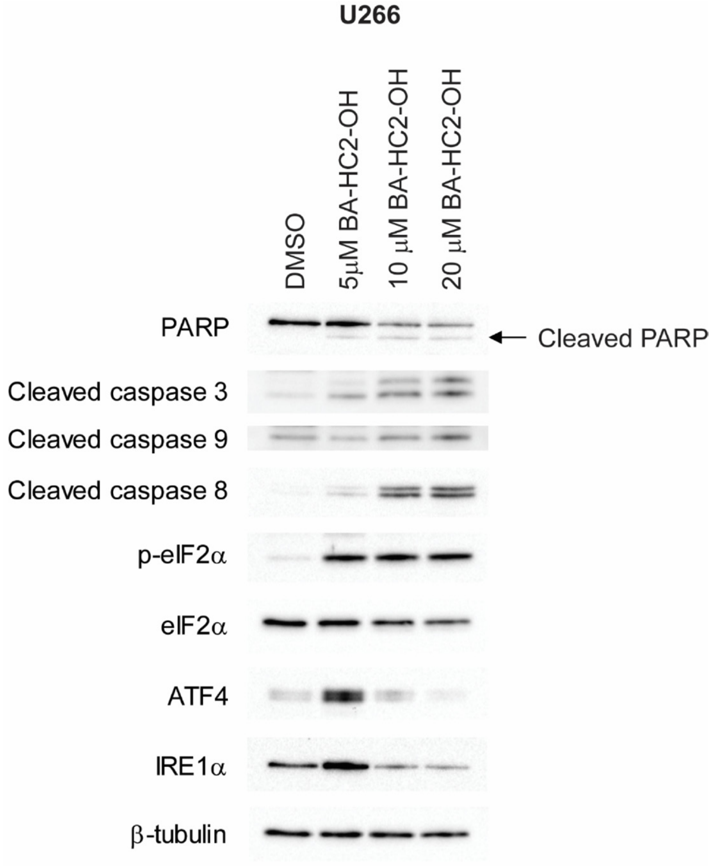

Supplementary Figure 7: BA-HC2-OH induces markers of apoptosis and ER stress/UPR. U266 cells were incubated for 48 hours in the presence or absence of DMSO (solvent control) or BA-HC2-OH (5 μM, 10 μM or 20 μM). Immunoblot analysis of PARP, cleaved caspases-3, -8 and -9, phosphorylated eIF2α (p-eIF2α), eIF2α, ATF4, and IRE1α was performed along with β-tubulin as a loading control.

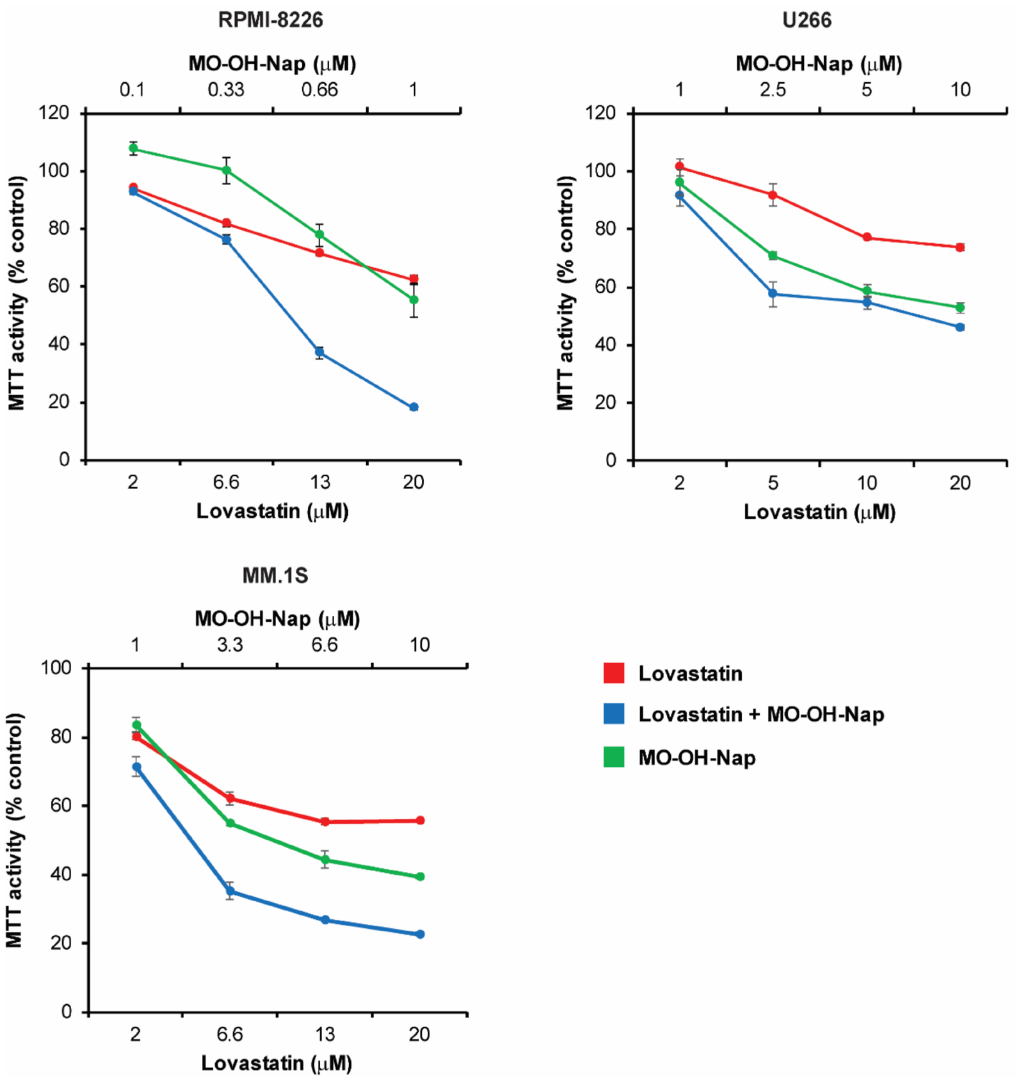

Supplementary Figure 8: Interaction between MO-OH-Nap and lovastatin. Three myeloma cell lines were incubated in the presence or absence of MO-OH-Nap and/or lovastatin. MTT assays were performed. Data are expressed as percentage of control (mean ± standard deviation, *n* = 4).

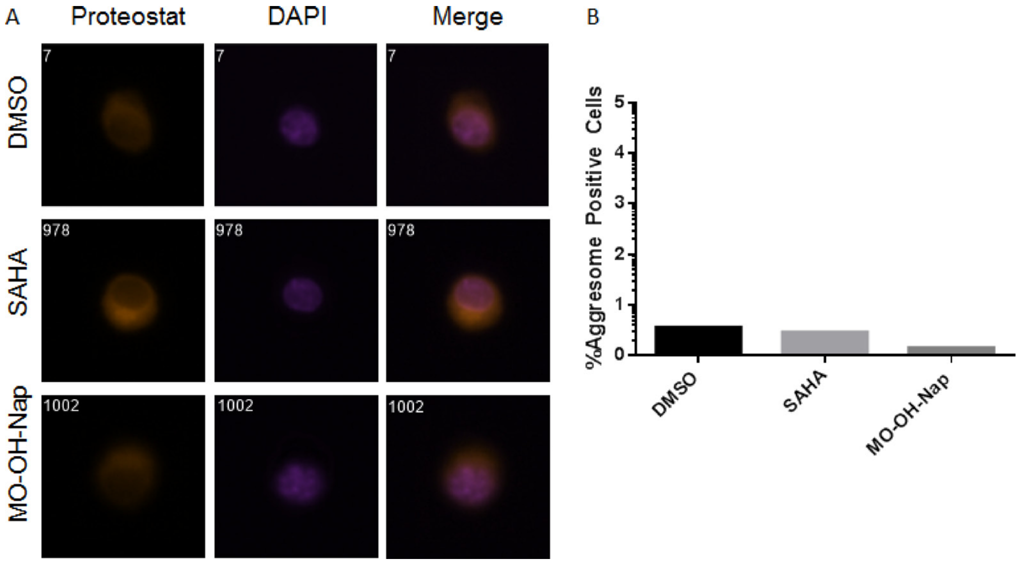

Supplementary Figure 9: MO-OH-Nap does not impact aggresome formation. U266 cells were treated for 24 hours with 1 µM SAHA or 10 µM MO-OH-Nap. Aggresome staining was performed as described in the Materials and Methods. Cells were gated as aggresome-positive for fluorescence intensities that were low Proteostat intensity in the nucleus and high Proteostat intensity outside the nucleus, indicating a perinuclear aggresome versus aggregates dispersed throughout the cell. Representative cells are shown in (**A**) and the analyzed data in (**B**).

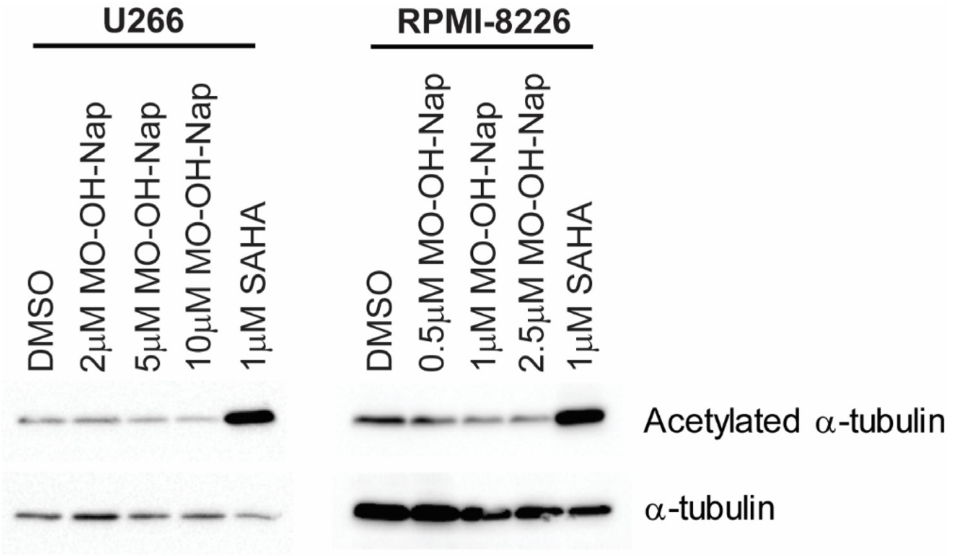

Supplementary Figure 10: Incubation with MO-OH-Nap does not alter levels of acetylated alpha-tubulin. U266 and RPMI-8226 cells were incubated for 48 hours in the presence or absence of DMSO (solvent control), MO-OH-Nap or SAHA (positive control). Immunoblot analysis of acetylated alpha-tubulin and alpha-tubulin (loading control) was performed.

Supplementary Table 1: Antibodies used for immunoblot analysis

	**Primary Antibody Source (catalog number) (dilution)**	**Secondary Antibody Source (catalog number) (dilution)**
Acetylated-alpha tubulin	Cell Signaling (5335T) (1:1000)	Amersham (NA934) (1:1000)
Alpha-tubulin	Abcam (EP1332Y) (1:25,000)	Amersham (NA934) (1:1000)
ATF-4	Cell Signaling Technology (11815) (1:1000)	Amersham (NA934) (1:1000)
Acetyl-Histone H4 (Lys8)	Cell Signaling Technology (2594) (1:1000)	Amersham (NA934) (1:1000)
Acetyl-Histone H3 (Lys9)	Cell Signaling Technology (9469) (1:3000)	Amersham (NA934) (1:1000)
Acetyl-Histone H3 (Lys23)	Cell Signaling Technology (14932) (1:1000)	Amersham (NA934) (1:1000)
Cleaved caspase 3	Cell Signaling Technology (9664) (1:500)	Amersham (NA934) (1:1000)
Cleaved caspase 9	Cell Signaling Technology (9501) (1:500)	Amersham (NA934) (1:1000)
Cleaved caspase 8	Cell Signaling Technology (9496) (1:500)	Amersham (NA934) (1:1000)
eIF2α	Cell Signaling Technology (9722) (1:1000)	Amersham (NA934) (1:2000)
Histone H3	Cell Signaling Technology (9715S)	Amersham (NA934) (1:1000)
Histone H4	Abcam (177840)	Amersham (NA934) (1:1000)
Phospho-eIF2a	Cell Signaling Technology (3597) (1:1000)	Amersham (NA934) (1:1000)
IRE1α	Cell Signaling Technology (3294) (1:2000)	Amersham (NA934) (1:2000)
PARP	Santa Cruz (sc-7150) (1:10,000)	Amersham (NA934) (1:2000)
PERK	Santa Cruz (sc-13073) (1:500)	Amersham (NA934) (1:1000)
β-tubulin	Sigma (T5201) (1:40000)	Amersham (NA931) (1:1000)

All primary and secondary antibodies used for western blot analysis are shown. All primary antibodies were incubated at 4°C overnight. All second antibodies were incubated at room temperature for one hour**.**

Supplementary Table 2: Ingenuity pathway analysis: upstream analysis

MOOH-24 hr vs Control			
**Molecule**	**Prediction**	**z-score**	***p*-value**
HIF1A	Activated	3.944	1.38E-21
TP53	Activated	1.956	2.15E-18
ATF4	Activated	3.251	1.17E-16
EPAS1	Activated	2.55	1.06E-15
IL2	Activated	1.854	2.24E-15
Hdac	Inhibited	−2.905	6.43E-05
MOOH-48hr vs Control			
**Molecule**	**Prediction**	**z-score**	***p*-value**
XBP1	Activated	4.371	9.28E-27
ATF4	Activated	4.214	6.19E-24
TP53	Activated	2.29	1.31E-23
EIF2AK3	Activated	2.099	1.02E-20
ATF6	Activated	2.275	8.88E-12
Hdac	Inhibited	−3.359	5.53E-50
Saha-24hr vs Control			
**Molecule**	**Prediction**	**z-score**	**p-value**
TP53	Activated	4.704	9.26E-24
MYC	Inhibited	−3.844	6.46E-19
CDKN1A	Activated	2.167	9.18E-16
TGFB1	Activated	3.028	1.21E-15
TBX2	Inhibited	−2.867	1.79E-15
Hdac	Inhibited	−2.737	1.19E-04

Differentially expressed genes were analyzed using Ingenuity Pathway Analysis. The top hit for "Upstream Analysis" are shown, as well as their predicted activation or inhibition and the associated Z-score and *p*-value of the analysis. A z-score > $|1.5|$ and a *p*-value < 0.05 were considered significant.

Supplementary Table 3: Combination indices from isobologram analysis of MTT cytotoxicity assays

	**RPMI-8226**	**U266**	**MM.1S**
**Bortezomib + MO-OH-Nap**	IC_{30}: 0.45<br>IC_{50}: 0.72	IC_{30}: 0.65<br>IC_{50}: 0.73	IC_{30}: 0.32<br>IC_{50}: 0.69
**Lovastatin + MO-OH-Nap**	IC_{30}: 0.56<br>IC_{50}: 0.31	IC_{30}: 1.0<br>IC_{50}: 1.2	IC_{30}: 0.84<br>IC_{50}: 0.63

Combination indices (CI) for the IC_{30} and IC_{50} values for MM cells treated with either a combination of bortezomib + MO-OH-Nap or Lovastatin + MO-OH-Nap are shown. As per Chou and Talalay, a CI <1 represents a synergistic interaction, CI = 1 represents an additive interaction and CI >1 represents an antagonistic interaction.

Supplementary Table 4: Primer sequences used for XBP-1 PCR and qRT-PCR

	Forward:	Reverse:
**CHOP**	TCTTCACCACTCTTGACCCTGCTT	GTTCTTTCTCCTTCATGCGCTGCT
**β-actin**	ACGTTGCTATCCAGGCTGTGCTAT	TTAATGTCACGCACGATTTCCCGC
**XBP-1**	GTTGAGAACCAGGAGTTAAGACAG	CAGAGGGTATCTCAAGACTAGG
